# Herbal components of Japanese Kampo medicines exert laxative actions in colonic epithelium cells via activation of BK and CFTR channels

**DOI:** 10.1038/s41598-019-52171-z

**Published:** 2019-10-29

**Authors:** Tomohiro Numata, Kaori Sato-Numata, Yasunobu Okada

**Affiliations:** 1 Department of Physiology, Graduate School of Medical Sciences, Fukuoka University, Fukuoka, 814-0180 Japan; 2 Japan Society for the Promotion of Science, Tokyo, 102-0083 Japan; 3 Department of Physiology, Kyoto Prefectural University of Medicine, Kyoto, 602-8566 Japan; 4 National Institute for Physiological Sciences, Okazaki, 444-8585 Japan

**Keywords:** Cell biology, Gastroenterology

## Abstract

Japanese Kampo medicines Junchoto and Mashiningan are mixtures of numerous herbal plant extracts and empirically known to exert laxative actions by stimulating fluid secretion in the colonic epithelium. However, it is unknown which and how the herbal components of these crude Kampo drugs are effective to stimulate ion effluxes causing fluid secretion. Here, we selected four herbal components of Junchoto and Mashiningan, Mashinin (MSN), Kyonin (KYN), Tonin (TON), and Daio (DIO), which are putatively laxatives, and examined their effects on the ion channel activity of human colonic epithelial Caco-2 cells. Patch clamp analyses revealed that MSN activated whole-cell current characteristics of the cystic fibrosis transmembrane conductance regulator (CFTR) channel, whereas KYN, TON, and DIO activated the large-conductance and voltage-activated K^+^ (BK) channel. Furthermore, electronic cell sizing showed that MSN induced secretory volume decrease (SVD) sensitivity to a CFTR blocker, whereas TON, KYN, and DIO induced SVD sensitivity to a K^+^ channel blocker. In conclusion, MSN and TON, KYN, and DIO promote fluid secretion from colonic epithelial cells by activating CFTR and BK channels. Thus, Japanese Kampo medicines, Junchoto and Mashiningan, exert anti-constipation actions by inducing KCl efflux through the combined actions of CFTR- and BK-stimulating herbal components.

## Introduction

Chronic constipation is an extremely common gastrointestinal disorder that severely affects quality of life. Its prevalence increases with age^1,2^ and may reach about 80% in nursing home residents^3^. Current management of constipation requires a combination of laxatives for patients that do not respond to basic treatment by lifestyle modifications^4^.

The types of laxatives are mainly classified by the mechanism of action as follows. *Bulk-forming laxatives* retain fluid, thereby increasing the bulk of stools. *Osmotic laxatives* contain non-absorbable molecules that drive water from the intestinal epithelium. *Stimulant laxatives* stimulate nerves, muscles, and their regulatory receptors to increase bowel movements. *Stool-softener laxatives* lead to water and fats penetrating into the stool or coat the intestinal surface with oils, thereby making stools easier to pass. *Secretagogue laxatives* induce electrolyte secretion across the intestinal epithelium, driving intestinal fluid secretion. The most notable and recent secretagogue type of laxative is lubiprostone^5,6^ that was initially reported to activate ClC-type anion channel ClC-2^7^, but is now well established to activate a cyclic AMP-activated anion channel, cystic fibrosis transmembrane conductance regulator (CFTR)^6,8^.

Traditional Japanese Kampo medicines Junchoto (JCT) and Mashiningan (MSG) have been recently shown to induce cAMP production in the intestinal epithelium and activate CFTR Cl^−^ channels, thereby inducing intestinal fluid secretion^9,10^. Kampo medicines are mixtures of numerous herbal plant extracts. However, it is often undetermined which herbal components exert secretagogue actions as potent laxatives.

JCT consists of 10 herb plant extracts including *Rhei Rhizoma* (rhubarb in English or Daio in Japanese), *Cannabis Fructus* (hemp fruit or Mashinin), *Armeniacae Semen* (apricot kernel or Kyonin), *Persicae Semen* (peach kernel or Tonin), *Magnoliae Cortex* (magnolia bark or Koboku), *Aurantii Fructus Immaturus* (immature orange or Kijitsu), *Glycyrrhizae radix* (glycyrrhiza or Kanzo), *Rehmanniae Radix* (rehmannia root or Jio), *Angelicae Acutilobae Radix* (Japanese angelic root or Toki), and *Scutellariae Radix* (scutellaria root or Ogon). MSG is the mixture of 6 herb plant extracts including *Rhei Rhizoma*, *Cannabis Fructus*, *Armeniacae Semen*, *Magnoliae Cortex*, *Aurantii Fructus Immaturus*, and *Paeoniae Radix* (poeny root or Shakuyaku). Among them, Daio (DIO), Mashinin (MSN), and Kyonin (KYN), which are commonly contained in JCT and MSG, and Tonin (TON), a component of JCT, but not MSG, are empirically known to have laxative effects.

Therefore, the present study aimed to determine whether these four herbal mixtures exert secretory actions and the mechanisms underlying their secretory actions in colonic epithelial cells.

## Results

### Four herbal components of Japanese Kampo laxatives stimulate fluid secretion by activating whole-cell ionic conductance in Caco-2 cells

**Figure 1:**
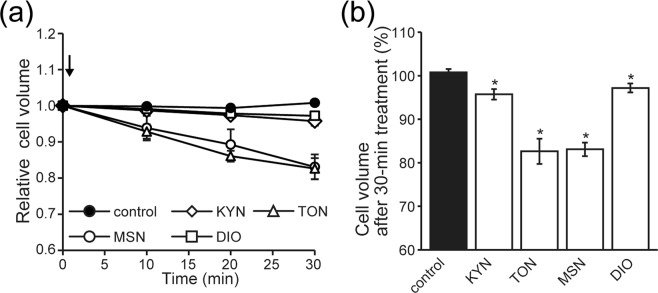
Induction of the secretory volume decrease (SVD) in human colonic epithelial Caco-2 cells by four herbal components in Japanese Kampo laxative medicines Junchoto and Mashiningan. (**a**) Time courses of changes in the mean cell volume. At time 0, Kyonin (KYN), Tonin (TON), Masinin (MSN), or Daio (DIO) was applied at 400 μg/ml except under the control condition. SVD events were monitored by the electronic sizing technique. (**b**) Percentage of the cell volume after 30 min of treatment with each herbal component relative to the initial cell volume. Each column represents the mean ± S.E.M. (n = 5). *P < 0.05 compared with the control volume.

Solute secretion from cells drives the transfer of osmotically obliged water, resulting in a cell volume reduction, which is called a secretory volume decrease (SVD)^10,11^. We therefore investigated herbal components in two types of Japanese Kampo medicines, JCT and MSG, which are widely used as laxatives, by measuring the SVD in human colonic epithelial Caco-2 cells. As shown in Fig. 1, exposure of Caco-2 cells to either MSN or TON resulted in a marked SVD by ~17% of the control cell volume at 30 min after administration. In contrast, applications of KYN and DIO induced smaller, but significant, SVD events by ~4% and ~3% of the control cell volume at 30 min after administration, respectively.

**Figure 2:**
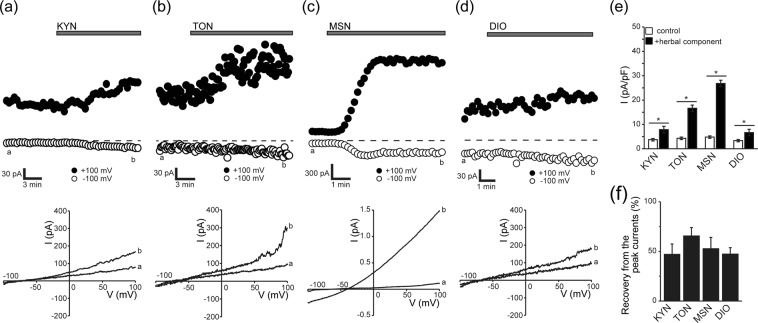
Activation of whole-cell membrane conductance evoked by KYN, TON, MSN, or DIO in Caco-2 cells under a nystatin-perforated patch-clamp. (**a**–**d**) Responses of whole-cell currents to stimulation by KYN, TON, MSN, or DIO, respectively. Top panels: Representative time courses of changes in whole-cell currents recorded at −100 mV (open circles) and +100 mV (filled circles) before and after application of each herbal component at 400 μg/ml during application (every 10 s) of ramp pulses of −100 to +100 mV from a holding potential of −60 mV. Bottom panels: Corresponding *I*-*V* relationships recorded at time points *a* and *b* in the top panels. The reversal potentials of these herbal component-induced currents were determined from the intersection of the *I-V* curves obtained in the absence and presence of a herbal component: KYN (−70.6 ± 1.7 mV, n = 7), TON (−72.5 ± 1.0 mV, n = 9), DIO (−73.2 ± 1.6 mV, n = 5), MSN (−40.1 ± 1.9 mV, n = 5). (**e**) Peak current densities measured at +100 mV before (control) and after (+herbal component) application of KYN, TON, MSN, and DIO. Each column represents the mean ± S.E.M. (n = 5–8). *P < 0.05 compared with the control value. (**f**) Percent recovery from peak whole-cell currents at 15 min after washout of the herbal component (KYN, TON, MSN, or DIO) calculated by the equation: % Recovery = 100 − (100 × [(*I*_2_ − *I*_1_)/(*I*_3_ − *I*_1_)], where *I*_1_ is the amplitude of the control current at +100 mV before herbal component application, *I*_2_ is the peak current attained after application of the herbal component, and *I*_3_ is the current measured at 15 min after washout of the herbal component. Each column represents the mean ± S.E.M. (n = 5–7).

Because isosmotic fluid secretion associated with SVD must be driven by ion fluxes, we next examined the effects of these four herbal components on whole-cell membrane conductance in Caco-2 cells under a nystatin-perforated patch-clamp. As shown in Fig. 2a–d (top panels) and summarized in Fig. 2e, all of these herbal components gradually increased the membrane conductance, and the effect was partially reversible within 15 min after washout of herbal components (Fig. 2f). The current-voltage (*I-V*) relationships measured by a ramp clamp before and after exposure to herbal components were found to intersect at −70 to −75 mV for KYN, TON, and DIO (Fig. 2a,b,d, bottom panels), whereas the *I-V* curves intersected at around −40 mV for MSN (Fig. 2c, bottom panel). Because the reversal potentials of K^+^ and Cl^−^ are −82 and −41 mV, respectively, these results strongly suggest that MSN predominantly activates Cl^−^ conductance, whereas KYN, TON, and DIO mainly activate K^+^ conductance in Caco-2 cells.

### Mashinin activates CFTR channels endogenously expressed in Caco-2 cells and heterologously expressed in HEK293T cells

**Figure 3:**
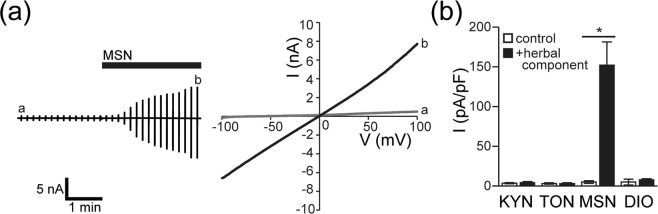
Activation of whole-cell Cl^−^ currents evoked by MSN, but not by KYN, TON, or DIO, in HEK/CFTR cells under a conventional whole-cell patch-clamp. (**a**) Representative responses of whole-cell currents to MSN. Left panel: Time courses of changes in whole-cell currents before and after application of MSN (400 μg/ml) during application (every 10 s) of ramp pulses of −100 to +100 mV from a holding potential of 0 mV. (**b**) Corresponding *I*-*V* relationships recorded at time points *a* and *b* in the left panel. (**c**) Peak current densities recorded at +100 mV before (control) and after (+herbal component) application of each herbal component (400 μg/ml). Each column represents the mean ± S.E.M. (n = 5–10). *P < 0.05 compared with the control value.

**Figure 4:**
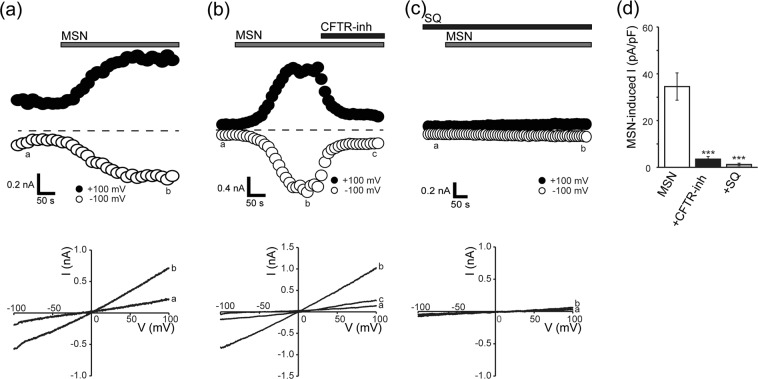
Activation of whole-cell CFTR currents evoked by MSN in Caco-2 cells under a conventional whole-cell patch-clamp. (**a**–**c**) Representative responses of whole-cell currents to MSN (**a**) and effects of treatment with 10 μM CFTR inhibitor-172 (CFTR-inh: **b**) and pretreatment with 100 μM SQ22536 (SQ: **c**). Top panels: Time courses of MSN-evoked whole-cell currents recorded at +100 mV (filled circles) and −100 mV (open circles) under a ramp clamp. Grey bars show application of 400 μg/ml MSN. Filled bars show application of CFTR-inh and SQ. Bottom panels: Corresponding *I*-*V* relationships recorded at time points *a*–*c* in the top panels. (**d**) Peak current densities measured at +100 mV in response to MSN alone and together with CFTR-inh or SQ. Each column represents the mean ± S.E.M. (n = 5–17). ***P < 0.001 compared with MSN alone.

Previous studies performed by short-circuit current measurements with Ussing chambers suggested that MSG and JCT produce membrane currents sensitive to a CFTR blocker, CFTR inhibitor-172 (CFTR-inh)^12,13^, across the polarized human bronchial epithelial cell layer^9,14^. In addition, we previously showed that JCT facilitates intestinal Cl^−^ secretion by activation of CFTR^10^. We therefore next investigated the effects of these four herbal components on CFTR activity in HEK/CFTR cells by conventional whole-cell recordings. As shown in Fig. 3, MSN activated Cl^−^ currents (a, left panel) exhibiting a linear *I-V* relationship (a, right panel) under symmetrical Cl^−^ conditions, whereas KYO, TON, and DIO did not activate whole-cell currents in HEK/CFTR cells (b). Similar Cl^−^ currents were also activated by MSN in Caco-2 cells (Fig. 4a). The MSN-activated Cl^−^ currents were found to be virtually abolished by treatment with CFTR-inh (Fig. 4b,d) and pretreatment with adenylate cyclase inhibitor SQ22536 (SQ; Fig. 4c,d). These results indicate that MSN, but neither KYN, TON, nor DIO, activates endogenous CFTR channels in Caco-2 cells.

### Kyonin, Tonin and Daio activate endogenous BK channels in Caco-2 cells

**Figure 5:**
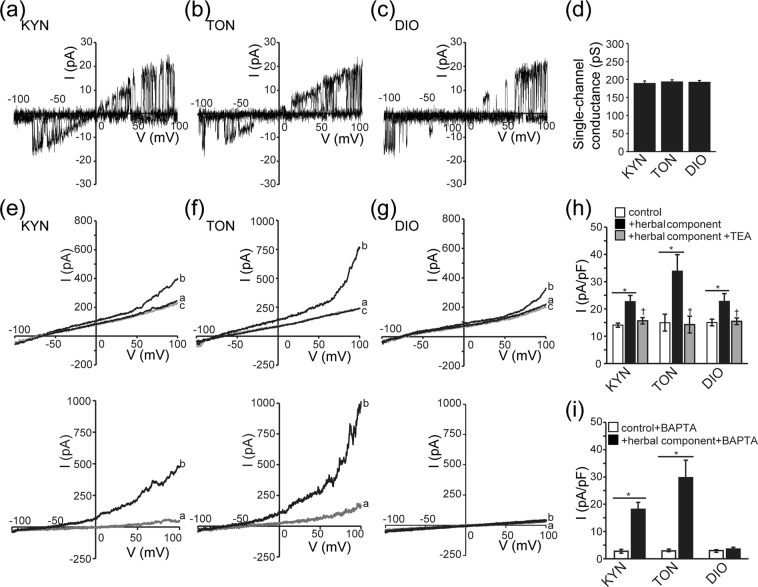
Activation of whole-cell and single-channel K^+^ currents in Caco-2 cells evoked by KYN, TON, and DIO. (**a**–**c**) *I-V* relationships of current responses to each herbal component (400 μg/ml) recorded upon application of ramp pulses of −100 to +100 mV from a holding potential of −60 mV under the indicated conditions. Single-channel events observed during application of ramp pulses in the cell-attached mode of a patch-clamp in a high K^+^ bath solution. Representative single-channel currents were observed only when a small number of channels were activated at the beginning of stimulation by each herbal component. (**d**) Single-channel conductance calculated by the slope of the *I*-*V* curves from the reversal potential to +100 mV. Each column represents the mean ± S.E.M. (n = 5). (**e**–**g**) Top panels: Inhibitory effects of 5 mM TEA on KYN-, TON- and DIO-induced whole-cell currents. *I*-*V* relationships were recorded upon application of ramp pulses before (*a*) and after stimulation with each herbal component alone (*b*) or together with TEA (*c*). Bottom panels: Effects of intracellular dialysis with 5 mM BAPTA on KYN-, TON- and DIO-induced whole-cell currents. *I*-*V* relationships were recorded upon application of ramp pulses before (*a*) and after stimulation with each herbal component alone (*b*). (**h**) Peak current densities recorded at + 100 mV before (white columns) and after application of each herbal component in the absence (black columns) and presence (grey columns) of TEA. Each column represents the mean ± S.E.M. (n = 5–6). *P < 0.05 compared with the control value. ^†^*P* < 0.05 compared with the herbal component-stimulated value in the absence of TEA. (**i**) Peak current densities recorded in the presence of intracellular BAPTA (5 mM) at +100 mV before (white columns) and after (black columns) application of herbal components. Each column represents the mean ± S.E.M. (n = 5–6). *P < 0.05 compared with the control value.

Because the data shown in Figs 2 and 3 suggest that KYN, TON, and DIO activate some K^+^ channels, but not CFTR Cl^−^ channels, we next analysed the properties of KYN-, TON- and DIO-activated currents in Caco-2 cells by cell-attached single-channel and nystatin-perforated whole-cell patch-clamp techniques. As shown in Fig. 5a–c, single-channel events were observed on Caco-2 cells only after stimulation with KYN, TON, or DIO. The unitary conductance evaluated by considering the reversal potentials was around 200 pS, as summarized in Fig. 5d. Under the high-input-resistance nystatin-perforated patch-clamp attained at the initial stage just after the giga-seal formation with nystatin-containing patch pipettes, similar single-channel events with unitary conductance of around 200 pS were observed frequently on Caco-2 cells stimulated with KYN, TON, or DIO (Supplementary Fig. S1). As shown in Fig. 5e–g (top panels) and summarized in Fig. 5h, KYN, TON, and DIO activated outwardly rectifying currents (curve *b*) with superimposing on background basal currents (curve *a*). The activated currents again exhibited voltage-dependent (depolarization-induced) activation and the reversal potential of around –70 to –80 mV. A well-known K^+^ channel blocker, tetraethylammonium (TEA), was found to abolish activation of KYN-, TON- and DIO-induced whole-cell currents in Caco-2 cells at 5 mM (curve *c*). Under intracellular Ca^2+^ chelation attained by introduction of BAPTA (5 mM) into the cells, KYN and TON were still effective to activate outwardly rectifying currents (Fig. 5e,f, bottom panels), but DIO did not activate the current (Fig. 5g, bottom panel), as summarized in Fig. 5i. These data indicate that KYN, TON, and DIO activate large-conductance and voltage-activated K^+^ (BK) channels in Caco-2 cells, and that DIO and KYN or TON induce activation of the channels in a manner directly dependent on and independent of intracellular Ca^2+^, respectively.

### Activation of CFTR and BK channels is responsible for fluid secretion in Caco-2 cells induced by herbal components of Japanese Kampo laxatives

**Figure 6:**
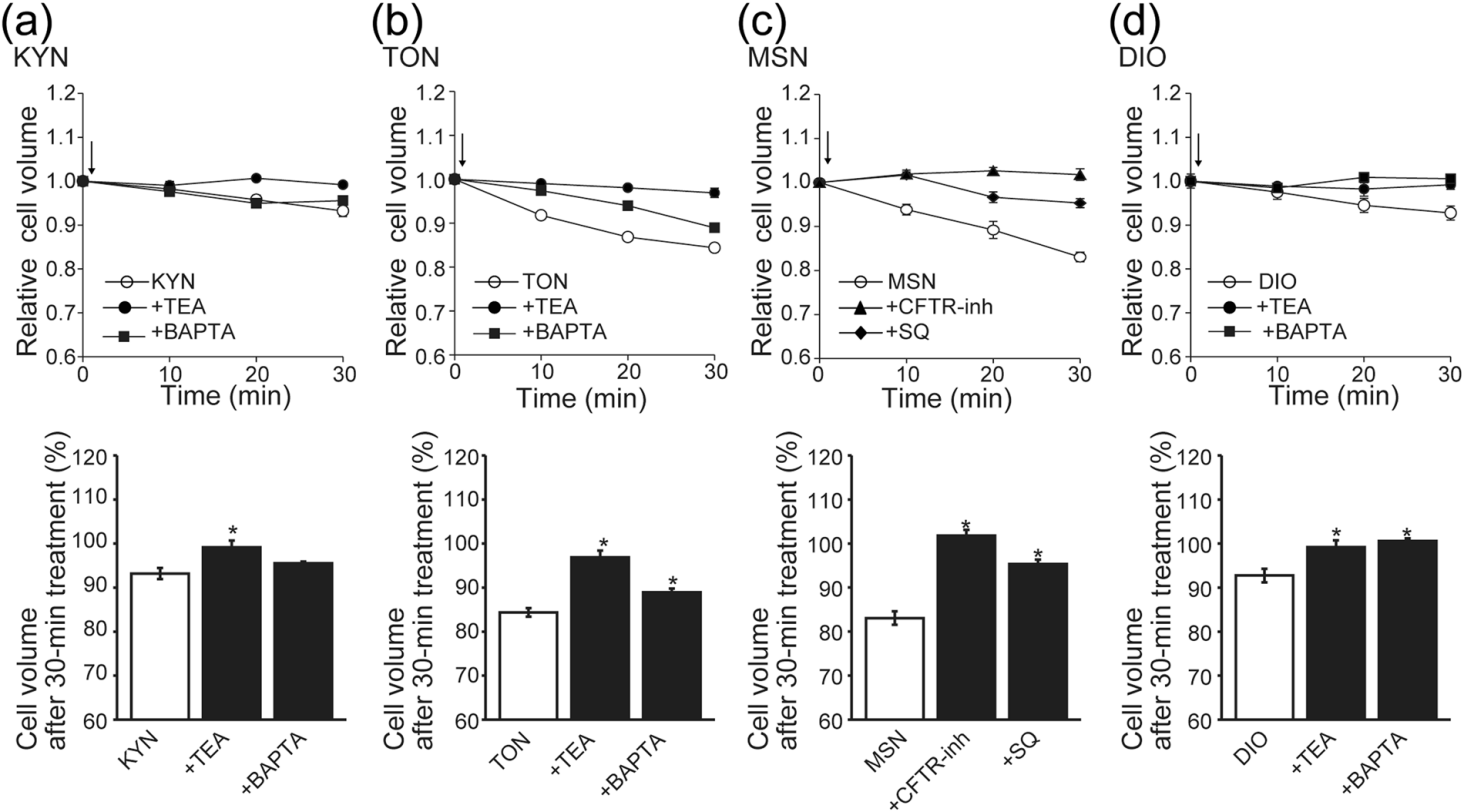
Sensitivity of SVD to TEA induced by KYN (**a**), TON (**b**), and DIO (**d**) and sensitivity of MSN-induced SVD to CFTR-inh or SQ22536 (**c**) in Caco-2 cells. Top panels: Time courses of changes in the mean cell volume. At time 0, each herbal component (400 μg/ml) was applied alone (open circles) or together with 5 mM TEA (filled circles), 10 μM CFTR-inh (filled triangles), or 100 μM SQ22536 (filled diamonds). Bottom panels: Percentage of the cell volume after 30 min of treatment relative to the initial cell volume. Each column represents the mean ± S.E.M. (n = 5). *P < 0.05 compared with the data of each herbal component alone.

Finally, we determined whether induction of SVD by the herbal components is caused by their activating actions on CFTR and BK channels. As shown in Fig. 6, KYN-, TON- and DIO-induced SVD events in Caco-2 cells were virtually eliminated by TEA (a, b and d, +TEA), whereas the MSN-induced SVD was prominently inhibited by SQ and abolished by CFTR-inh (c, +SQ and +CFTR-inh). Intracellular Ca^2+^ chelation completely inhibited DIO-induced SVD, but did not affect KYN-induced SVD and only partially suppressed TON-induced SVD (Fig. 6, +BAPTA).

Taken together, fluid secretion from Caco-2 cells in response to MSN is induced by activation of CFTR channels, and that in response to KYN, TON, or DIO is induced by BK channel activation. Thus, KCl efflux attained by parallel activation of BK and CFTR channels is responsible for fluid secretion induced by these herbal components of Japanese Kampo laxatives, Junchoto and Mashiningan.

## Discussion

In response to stimulation by a variety of secretagogues, cell shrinkage is induced in association with fluid secretion in a number of secretory epithelial cell types (see Review^15^), which is called the secretory volume decrease (SVD)^11^. In the present study, four herbal plant extracts, hemp fruit MSN, apricot kernel KYN, peach kernel TON, and rhubarb DIO, which are putatively known to exert laxative effects, provoked the SVD response in human colonic epithelial Caco-2 cells (Fig. 1). The MSN-induced SVD in Caco-2 cells was suppressed by a CFTR blocker and adenylate cyclase inhibitor, whereas the SVD responses to KYN, TON, and DIO were inhibited by a K^+^ channel blocker (Fig. 6). In fact, patch-clamp experiments showed that MSN induced activation of CFTR-mediated anion currents in Caco-2 and CFTR-expressing HEK293T (HEK/CFTR) cells, and that KYN, TON, and DIO did not activate CFTR currents in both cell types but activated large-conductance and voltage-activated K^+^ (BK) channel currents in Caco-2 cells (Figs 2–5). Taken together, MSN induces fluid secretion by stimulating CFTR anion channels through promotion of cAMP production, whereas KYN, TON, and DIO cause fluid secretion by activating BK channels in human colonic epithelial Caco-2 cells. In addition, among the four herbal extracts, MSN and TON exhibited much higher fluid-secreting activity estimated by the SVD extent than KYN and DIO.

Intracellular Ca^2+^ chelation by BAPTA (5 mM) introduced into the cells abolished DIO-induced activation of BK channels (Fig. 5g, bottom panel and i). In contrast, BK channels activated by KYN and TON were unaffected by this treatment (Fig. 5e,f, bottom panels and i). The BK channel is a homotetramer of the pore-forming α subunit and modulatory auxiliary subunits including four types of β subunits and four types of γ subunits (see Review^16^). Recently, a Ca^2+^-independent activation mechanism of BK channels was found to be mediated by their γ-subunits including LRRD26, LRRD52, LRRD55, and LRRD38 (see Reviews^16,17^). Thus, there is the possibility that KYN- and TON-induced activation is mediated by γ subunits of BK channels.

MSN is derived from hemp that has long been used as the raw material for fibres, foods, oils, and medicines. Hemp seed soft capsule (HSSC) ameliorates constipation by increasing the wet weight and water content of stool of loperamide-induced constipation model rats. This effect was suggested to be induced by activating CFTR, Ca^2+^-activated Cl^−^ channels, Na^+^-K^+^-2Cl^−^ (NKCC) cotransporters, and Cl^−^/HCO_3_^−^ exchangers in colonic epithelia, based on pharmacological sensitivity of HSCC-induced short-circuited currents in rat colonic mucosa^18^. Thus, our present study suggests that the CFTR-activating effect of HSSC is, at least in part, caused by MSN. However, there are no studies of the effect on the intestinal transport of the peach kernel, from which TON is derived, although its extract exhibits an anti-cancer action in human colonic cancer HT-29 cells^19^.

For airway and intestine epithelial Cl^−^ transport mediated by CFTR, TMEM16A (ANO1) is required for proper expression of CFTR in the plasma membrane^20^. TMEM16A is expressed in colonic Caco-2 cells and involved in glucose-induced enhancement of intestinal Cl^−^ secretion caused by rotavirus infection^21^. However, under the present experimental conditions, Ca^2+^-activated Cl^−^ currents were not observed. Thus, the activity of TMEM16A does not appear to be directly implicated in SVD stimulated by the herbal components, although there remains an indirect role of TMEM16A in plasmalemmal CFTR expression.

MSN consists of numerous chemical substances including fatty acids, such as linoleic acid, palmitic acid, oleic acid, and linolenic acid, as well as cannabicin A–G and vitamin E (https://www.genome.jp/kegg/). TON contains a much greater variety of chemical substances including fatty acids, such as palmitic acid, oleic acid, linolenic acid, and stearic acid, as well as steroids and amygdalin^22^. Thus, fatty acids, such as palmitic acid, oleic acid, and linolenic acid, which are commonly contained in MSN and TON, could be ruled out as CFTR- and BK-activating agents from the effective chemical components of MSN and TON, respectively. In fact, when we tested the change in CFTR currents in response to administration of 0.5 mM oleic acid, linolenic acid, or palmitic acid in Caco-2 cells, almost no current responses were observed (T. Numata: unpublished data, n = 6–11). Information on the chemical substances of the components in MSN, TON, KYN, and DIO is provided by the Japan Science and Technology Agency National Bioscience Database Center (http://togodb.biosciencedbc.jp/togodb/view/knapsack_kampo_syoyaku). Future studies are warranted to identify the chemical substances that are CFTR-activating MSN component(s) and BK-activating TON component(s).

## Methods

### Reagents

Dimethyl sulfoxide (DMSO), *O*,*O*′-bis(2-aminophenyl)ethyleneglycol-*N*,*N*,*N*′,*N*′-tetraacetic acid, tetrapotassium salt, hydrate (BAPTA), and BAPTA-AM were purchased from Dojindo Laboratories (Kumamoto, Japan). Tetraetylammonium chloride (TEA) was purchased from Tokyo Kasei Kogyo Company (Tokyo, Japan). CFTR inhibitor-172 and SQ22536 were obtained from Sigma-Aldrich (St. Louis, MO, USA). Mashinin (*Cannabis Fructus*), Kyonin (*Armeniacae Semen*), Tonin (*Persicae Semen*), and Daio (*Rhei Rhizoma*) were obtained from Nakaya Hikojyuro Pharmacy (Ishikawa, Japan). Mashinin, Kyonin, Tonin, and Daio powders were dissolved in DMSO at concentrations from 400 mg/ml and used on the same day.

### Cell culture and cDNA expression

Human colonic epithelial Caco-2 cells and human embryonic kidney epithelial HEK293T cells overexpressing CFTR (HEK/CFTR cells) were grown in Dulbecco’s modified Eagle’s medium supplemented with 10% fetal bovine serum, 30 U/ml penicillin, and 30 μg/ml streptomycin, at 37 °C with 5% CO_2_. To culture Caco-2 cells, 1% non-essential amino acids was added to the culture medium. Twenty-four hours after plating on culture dishes, HEK/CFTR cells were transfected with either a pCIneo-IRES-GFP vector or human CFTR-pCIneo-IRES-GFP vector (a generous gift from Dr. RZ Sabirov^23^). Lipofectamine 2000 (Invitrogen, Carlsbad, CA, USA) was used as the transfection reagent following the manufacturer’s instructions. Electrophysiological measurements were performed at 36–72 h after transfection. For cell volume measurements and electrophysiological experiments, Caco-2 and HEK/CFTR cells were dissociated using Accumax (Innovative Cell Technologies, Inc, San Diego, CA, USA), detached from culture dishes, and cultured in suspension with agitation for 15–300 min.

### Mean cell volume measurements

Cell volume was measured at room temperature by electronic sizing with a Coulter-type cell size analyser (CDA-500; Sysmex, Hyogo, Japan). The mean volume of the cell population was calculated from the cell volume distribution measured after the machine was calibrated with latex beads of a known volume. Isotonic Tyrode solution contained (in mM): 140 NaCl, 5 KCl, 1 MgCl_2_, 2 CaCl_2_, 10 D-glucose, and 10 HEPES (pH 7.4 adjusted by NaOH and osmolality adjusted to 300 mosmol/kg-H_2_O with D-mannitol). The relative cell volume was defined by the following equation: relative cell volume = *V*_*Test*_/*V*_*Ctl*_, where *V*_*Ctl*_ and *V*_*Test*_ are the mean cell volume before and after application of DMSO (control), MSN, TON, KYN, or DIO, respectively.

### Electrophysiology

Membrane currents of the cell were recorded at room temperature (22–27 °C) using the conventional and nystatin-perforated whole-cell modes of the patch-clamp technique with an Axopatch 200B patch-clamp amplifier (Axon Instruments/Molecular Devices, Union City, CA, USA). For whole-cell recordings, patch electrodes were prepared from borosilicate glass capillaries with an input resistance of 3–5 MΩ. Current signals were filtered at 5 kHz with a four-pole Bessel filter and digitized at 20 kHz. pCLAMP software (version 10.5.1.0; Axon Instruments/Molecular Devices) was used for command pulse control, data acquisition, and analysis. Data were also analysed using Origin software (OriginLab Corp., Northampton, MA, USA). For conventional whole-cell recordings, series resistance was compensated (to 70–80%) to minimize voltage errors. For CFTR current recordings, conventional whole-cell recordings were performed using the external solution containing (in mM) 110 CsCl, 2 CaCl_2_, 1 MgCl_2_, 5 glucose, and 10 HEPES (pH 7.4 adjusted by CsOH and osmolality adjusted to 310 mosmol/kg-H_2_O with D-mannitol), and the pipette solution containing (in mM) 110 CsCl, 2 MgSO_4_, 1 EGTA, 10 HEPES, 1 Na_2_ATP, and 15 Na-HEPES (pH 7.4 adjusted by CsOH, and osmolality adjusted to 300 mosmol/kg-H_2_O with D-mannitol). For whole-cell BK current recordings under intracellular Ca^2+^-chelating conditions, conventional whole-cell recordings were performed using isotonic Tyrode solution for external and pipette solutions, which contained (in mM) 55 K_2_SO_4_, 20 KCl, 1 MgCl_2_, 5 BAPTA, and 5 HEPES (pH adjusted to 7.4 by KOH and osmolality adjusted to 300 mosmol/kg-H_2_O with D-mannitol). For BK single-channel recordings, cell-attached recordings were obtained using high K^+^ Tyrode solution for the external solution, which contained (in mM) 5 NaCl, 140 KCl, 1 MgCl_2_, 2 CaCl_2_, 10 D-glucose, and 10 HEPES (pH 7.4 adjusted by KOH and osmolality adjusted to 310 mosmol/kg-H_2_O with D-mannitol), and the pipette solution that contained (in mM) 55 K_2_SO_4_, 20 KCl, 1 MgCl_2_, and 5 HEPES (pH adjusted to 7.4 by KOH and osmolality adjusted to 300 mosmol/kg-H_2_O with D-mannitol). For nystatin-perforated whole-cell current measurements, cells were exposed to isotonic Tyrode solution and dialyzed against the pipette solution containing (in mM) 55 K_2_SO_4_, 20 KCl, 5 MgCl_2_, 0.2 EGTA, and 5 HEPES (pH adjusted to 7.4 by KOH and osmolality adjusted to 300 mosmol/kg-H_2_O with D-mannitol) supplemented with 200 μg/ml nystatin.

### Statistical evaluation

All data are expressed as means ± S.E.M. Data for each condition were acquired from at least three independent experiments. Statistical analyses were performed using the Student’s *t*-test. *P* < 0.05 was considered as significant.

## Supplementary information

Supplementary Information

## Data Availability

All relevant data are included within the paper.
