# Supplementary material for: Herbal components of Japanese Kampo medicines exert laxative actions in colonic epithelium cells via activation of BK and CFTR channels
Source: Sci Rep. 2019 Oct 29;9:15554. doi: 10.1038/s41598-019-52171-z (PMC6820752; doi:10.1038/s41598-019-52171-z)
Supplement: Supplementary file 1 — Supplementary Information [file 41598_2019_52171_MOESM1_ESM.docx]

**Herbal components of Japanese Kampo medicines exert laxative actions in colonic epithelium cells via activation of BK and CFTR channels**

**Tomohiro Numata^1^*, Kaori Sato-Numata^1,2^ & Yasunobu Okada^3,4^**

^1^Department of Physiology, Graduate School of Medical Sciences, Fukuoka University, Fukuoka 814-0180, Japan. ^2^Japan Society for the Promotion of Science, Tokyo 102-0083, Japan. ^3^Department of Physiology, Kyoto Prefectural University of Medicine, Kyoto 602-8566, Japan. ^4^National Institute for Physiological Sciences, Okazaki 444-8585, Japan. *Correspondence and requests for materials should be addressed to T.N (email: numata@ fukuoka-u.ac.jp)


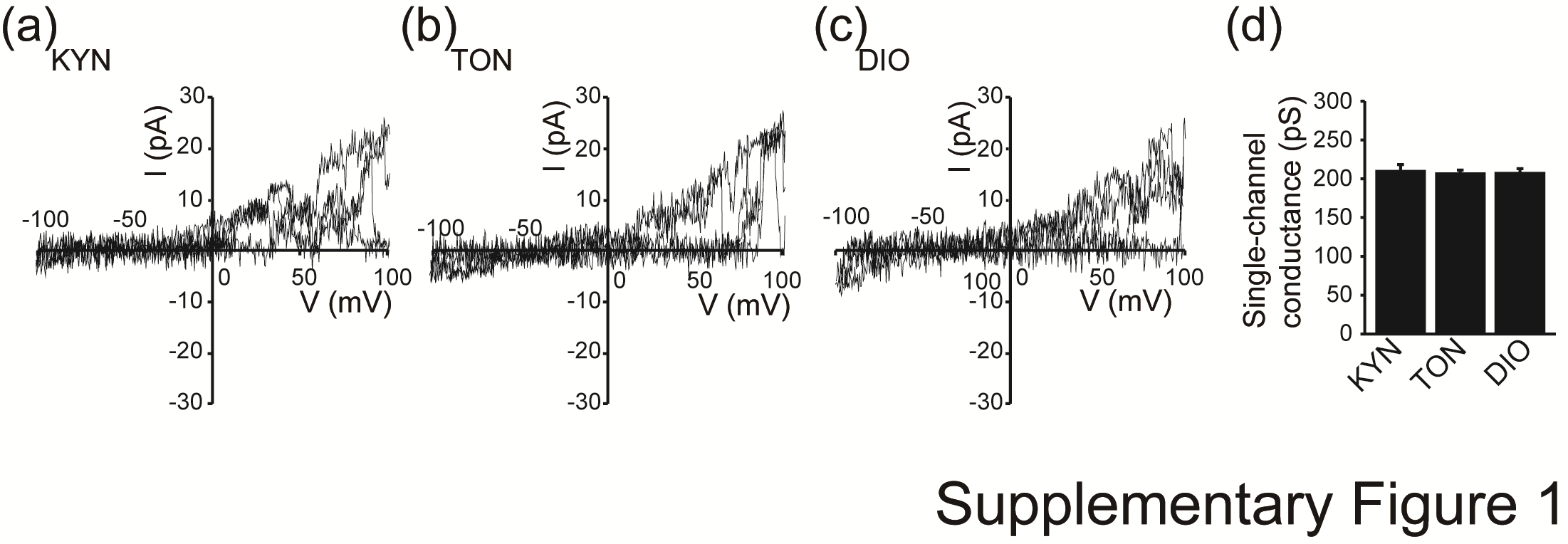


**Supplementary Figure 1.** Activation of single-channel K^+^ currents in Caco-2 cells evoked by Kyonin (KYN), Tonin (TON), and Daio (DIO). (**a–c**) *I-V* relationships of current responses to each herbal component (400 μg/ml) recorded upon application of ramp pulses of −100 to +100 mV from a holding potential of −60 mV. Single channel events observed at positive potentials during application of ramp pulses by the high input resistance nystatin-perforated patch-clamp are shown. Representative single-channel currents were observed only when a small number of channels were activated at the beginning of stimulation by each herbal component. Four sweeps of *I-V* curves are compositely presented. (**d**) Single-channel conductance calculated by the slope of *I*-*V* curves from the reversal potential to +100 mV. Each column represents the mean ± S.E.M. (n = 5–9).
